# Dietary Intervention for Overweight and Obese Adults: Comparison of Low-Carbohydrate and Low-Fat Diets. A Meta-Analysis

**DOI:** 10.1371/journal.pone.0139817

**Published:** 2015-10-20

**Authors:** Jonathan Sackner-Bernstein, David Kanter, Sanjay Kaul

**Affiliations:** 1 ExVivos, LLC, Hastings-on-Hudson, NY, United States of America; 2 Georgetown University Law Center, Washington, DC, United States of America; 3 Cedars-Sinai Medical Center, Los Angeles, CA, United States of America; University of Medicine & Dentistry of NJ—New Jersey Medical School, UNITED STATES

## Abstract

**Background:**

Reduced calorie, low fat diet is currently recommended diet for overweight and obese adults. Prior data suggest that low carbohydrate diets may also be a viable option for those who are overweight and obese.

**Purpose:**

Compare the effects of low carbohydrate versus low fats diet on weight and atherosclerotic cardiovascular disease risk in overweight and obese patients.

**Data Sources:**

Systematic literature review via PubMed (1966–2014).

**Study Selection:**

Randomized controlled trials with ≥8 weeks follow up, comparing low carbohydrate (≤120gm carbohydrates/day) and low fat diet (≤30% energy from fat/day).

**Data Extraction:**

Data were extracted and prepared for analysis using double data entry. Prior to identification of candidate publications, the outcomes of change in weight and metabolic factors were selected as defined by Cochrane Collaboration. Assessment of the effects of diets on predicted risk of atherosclerotic cardiovascular disease risk was added during the data collection phase.

**Data Synthesis:**

1797 patients were included from 17 trials with <1 year follow up in 12. Compared with low fat diet, low carbohydrate was associated with significantly greater reduction in weight (Δ = -2.0 kg, 95% CI: -3.1, -0.9) and significantly lower predicted risk of atherosclerotic cardiovascular disease events (p<0.03). Frequentist and Bayesian results were concordant. The probability of greater weight loss associated with low carbohydrate was >99% while the reduction in predicted risk favoring low carbohydrate was >98%.

**Limitations:**

Lack of patient-level data and heterogeneity in dropout rates and outcomes reported.

**Conclusions:**

This trial-level meta-analysis of randomized controlled trials comparing LoCHO diets with LoFAT diets in strictly adherent populations demonstrates that each diet was associated with significant weight loss and reduction in predicted risk of ASCVD events. However, LoCHO diet was associated with modest but significantly greater improvements in weight loss and predicted ASCVD risk in studies from 8 weeks to 24 months in duration. These results suggest that future evaluations of dietary guidelines should consider low carbohydrate diets as effective and safe intervention for weight management in the overweight and obese, although long-term effects require further investigation.

## Introduction

Historically, low-fat diets were advocated based on associations between dietary fat intake and cardiovascular risk[1] yet three lines of evidence suggest that low-fat diets might not be optimum for weight management. First, the Cochrane Collaboration review demonstrated over a decade ago that low-fat diets were not associated with clinically meaningful advantages in weight loss compared with caloric restriction after 6, 12 and 18 months.[2] Second, clinical trial evidence available in 1983 for the initial US and UK dietary guidelines did not demonstrate the superiority of low fat diets as first line for those overweight or obese.[3] And third, the large-scale randomized Women’s Health Initiative trial failed to show impact of low-fat diets on clinical outcomes,[4] with modest changes in lipid profiles and weight.[5,6]

In this context, the National Heart, Lung and Blood Institute convened an expert panel with the Obesity Society, the American Heart Association and the American College of Cardiology to address several questions, amongst which were the need to define the comparative efficacy of available diets for management of overweight and obesity in adults and to understand health effects of the resulting weight loss.[7]

To understand the potential efficacy and safety of dietary strategies, we performed a trial-level meta-analysis to compare the effects of low-carbohydrate (LoCHO) diets with low-fat (LoFAT) diets on weight loss and predicted cardiovascular risk in those who are overweight or obese. We synthesized the evidence via both classical frequentist and Bayesian methods.

## Methods

### Data Sources & Searches

The target population included the overweight and obese. Clinical trials were sought comparing LoCHO versus LoFAT diets. Pubmed.gov was searched for relevant trials with evaluation of citations in published literature reviews. (S1 File) Search strategy focused on randomized controlled trials (as well as systematic reviews and meta-analyses) evaluating weight management of the overweight and obese, using carbohydrate-restricted, fat-restricted or high protein diets.

### Study Selection

To be included requirements included: minimum of 8 weeks follow-up, LoFAT diet was defined to be at least as strict with respect to total fat consumption as in the Institute of Medicine’s report in 2002[8] (≤30% of calories from fat/day) and LoCHO diet was defined as total carbohydrate intake of 120 gm/day or less, recorded at least once during the study for each intervention. Trials were excluded if treatment allocation was not random, the population had comorbidities other than dyslipidemia, and/or participants included those ≤18 years of age. Trial selection criteria did not require documentation of ketosis in LoCHO group. Trials that included non-diet related lifestyle instructions in both intervention groups were eligible to be included.

### Data Extraction and Quality Assessment

Clinical variables were extracted (by DK and JSB) to enable characterization of the patient population enrolled in the individual trials. Data were abstracted for the endpoints (by JSB) as specified in the Cochrane Collaboration proposed protocol for the assessment of dietary interventions for the treatment of the overweight and obese, with the mean net change in weight chosen as the primary endpoint and the mean net changes in serum lipids and blood pressure selected as secondary endpoints,[9] which also enabled calculation of the predicted risk of cardiovascular events via the NHLBI Pooled Cohort equations.[10] Mean net change was calculated by subtracting mean change (from baseline to end of trial) in the LoFAT group from mean change in the LoCHO group (negative values favoring LoCHO and positive values favoring LoFAT group).

### Data Synthesis and Analysis

Predicted risk of atherosclerotic cardiovascular disease (ASCVD) events was determined using the Pooled Cohort Equations developed by NHLBI based on both ATP-3 and Framingham experiences. [10] These Pooled Cohort Equations were used to estimate the risk of fatal or nonfatal myocardial infarction or stroke, using mean values across trials for age, total cholesterol, HDL cholesterol and systolic blood pressure. As trials did not consistently report race/ethnicity, tobacco use or use of anti-hypertensive therapies, calculations were performed for Whites and African-Americans each with the following assumptions: no use of tobacco and no anti-hypertensive therapies (the lower-risk scenario); and 100% use of tobacco and 100% use of anti-hypertensive therapies (the higher-risk scenario).

Data were not extracted for types of fat or carbohydrates. Where variance was not reported for the change from baseline in any variable, it was calculated unless not possible, in which case the mean variance from the group of studies was imputed. Publications did not permit extraction of data on background medications or smoking status. To mitigate the risk of bias associated with different imputation methods, data were extracted preferentially for actual measurements without imputation, though trials that included imputation methods including last observation carried forward, with the goal of providing an intention-to-treat (ITT) analysis, were also included. Sensitivity analyses included assessment of the effects of the diets on weight for each group of trials separately (completer vs. ITT) along with other parameters of interest. Although we did not impute results, the data reported by others that included imputations were used in our analyses when data for completers was not provided. This approach limits the inferences and conclusions of these analyses to understanding the impact of these diets in those capable of adhering to the regimens.

### Statistical Analyses

Evidence was synthesized using both conventional frequentist and Bayesian approaches to meta-analysis using the random effects model to accommodate heterogeneity across the studies.

In the frequentist approach, outcome data were analyzed quantitatively using the R programming environment (v.3.03, r-project.org via R-Studio interface v.0.98.1060, rstudio.com) via the metafor package (v.1.9–4, r-project.org).[11] Mean differences (MD) along with 95% confidence interval (CI) were estimated and P values reported with two-sided significance tests.

The Bayesian approach utilized the random effects hierarchical model. Potential advantages of the Bayesian approach, including the appropriate reflection of the uncertainty in estimates of hyperparameters, have been previously described.[12] We used the open-source program OpenBUGS (Bayesian inference with Gibbs sampling) to fit the model using Markov Chain Monte Carlo (MCMC) sampling (v.3.2.2 rev 1063, openbugs.net). Posterior inferences (mean difference and 95% credible intervals [CrI]) were calculated by sampling from the posterior distribution of the parameters. We used non-informative priors (normal distribution with mean = 0, SD = 1.0E-6 for the overall mean difference and uniform distribution with a = 0 and b = 16 for the between study variance). In addition, the posterior probability that the difference was greater than 0 was estimated. Probabilities across a range of treatment effects were also computed.

The between group effects on the differences between predicted risk were assessed using t-tests for frequentist analyses in parallel with Bayesian modeling via the method of Kruschke[13] using BEST software, reported as mean with high density intervals, a correlate of 95% confidence intervals of frequentist and 95% credible intervals of Bayesian hierarchical methods [downloaded from http://www.indiana.edu/~kruschke/BEST/ on October 9, 2014], rjags for MCMC method (v.3-13, and JAGS (v.3.4.0, http://mcmc-jags.sourceforge.net/).

The database and software code for the meta-analyses are provided (S2 and S3 Files).

## Results

### Trials

**Fig 1:**
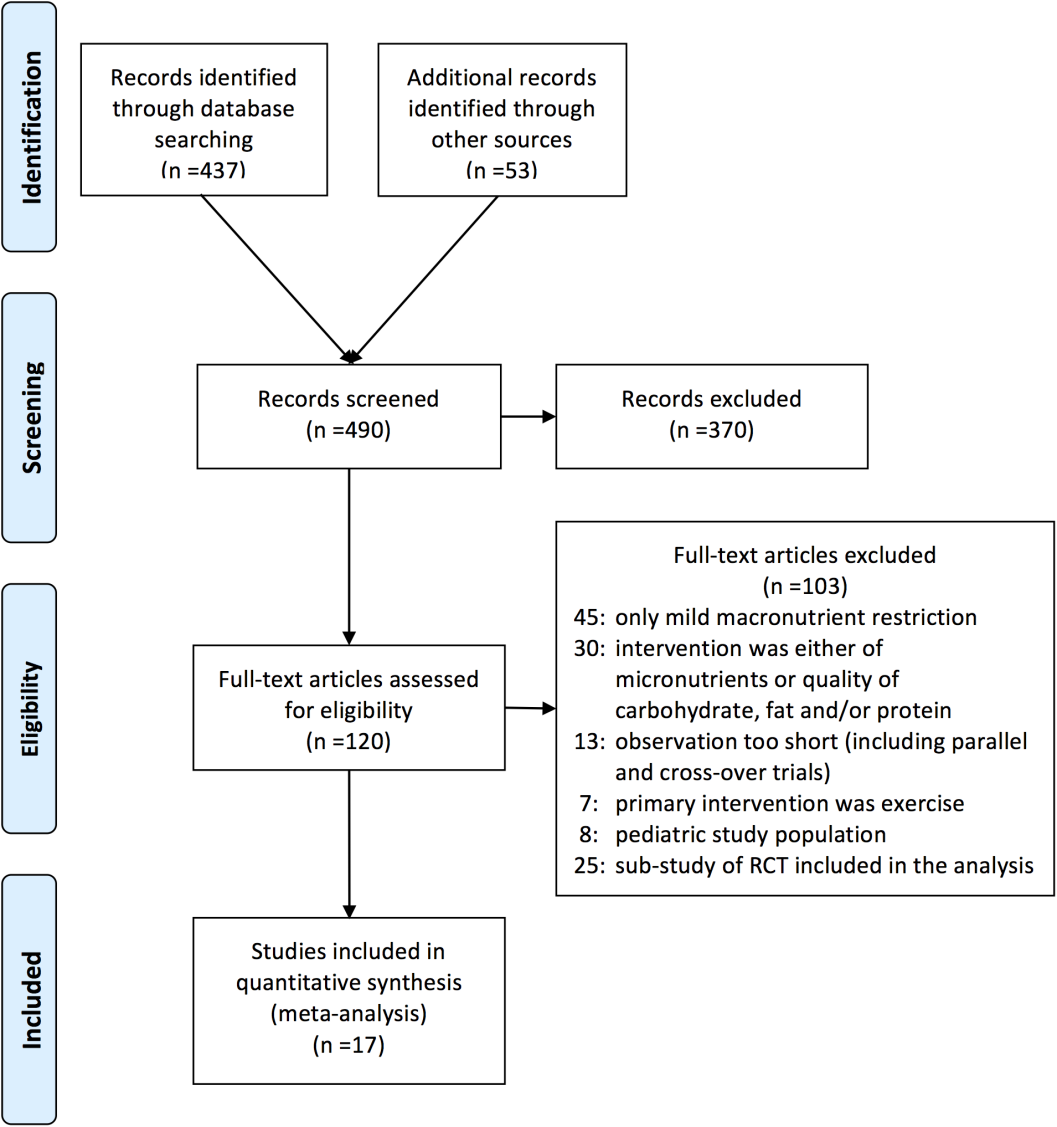
PRISMA Diagram. Identification and selection of RCTs.

The systematic literature search identified 490 articles, with 17 trials identified that met the inclusion criteria for this analysis. A total of 1,797 overweight and obese subjects (895 on LoCHO and 902 on LoFAT diets) were included.[14–30] Of 17 trials, 11 provided information for completers and 6 for ITT analyses. (Fig 1 and Tables 1 and 2) No trial that focused on patients with type 2 diabetes met criteria for inclusion in this analysis.

**Table 1:** Randomized clinical trials included in the meta-analyses: study characteristics.

Lead Author	Year	Calorie Restriction	Key Enrollment Criteria	Primary Endpoint(s)	Randomization	Outcomes Reported	Dropout	Study Power For Between Group Effect	Duration
Bradley	2009	500 kcal deficit per day in each group	BMI≥27	Insulin resistance (euglycemic-hyperinsulin-emic clamp)	Random number generator, blocked	Completers reported	Specifics not disclosed	Insulin Sensitivity	8 weeks
Brehm	2003	ad libitum LoCHO vs. calorie restricted LoFAT	BMI = 30–35	Weight and LDL	Specifics not disclosed	ITT with LOCF/BOCF with completers reported separately	Reasons listed	Specifics not disclosed	6 months
Brehm	2005	ad libitum LoCHO with ketosis vs. calorie restricted LoFAT	Women, BMI = 30–35	Resting energy expenditure	Randomization blocked, computer generated	Completers reported	Reasons listed	Specifics not disclosed	4 months
Brinkworth	2009	isocaloric with moderate energy restriction for both groups; Women~1429 & men~1667 kcal/d	Abdominal obesity + at least one risk factor for metabolic syndrome	Weight & Metabolic Effect	Specifics not disclosed	Completers reported	Reasons listed	Specifics not disclosed	1 year
Dansinger^a^	2005	None	BMI = 27–42 + at least one risk factor for metabolic syndrome	Weight	Computer generated, stratified	ITT (LOCF) with completers reported separately	Reasons listed	Weight at **Δ** = 3% (or **Δ**from baseline for each of 2%)	1 year
de Luis	2012	both calorie-restricted, ~1500kcal/day	BMI>30	Weight	Disclosed via envelope	All patients completed	All completed	Weight: power calculation stated differently in related publications	3 months
Flechter-Mors	2010	500 kcal deficit per day	metabolic syndrome, BMI = 27–45	Weight & body comp	Specifics not disclosed	Completers with ITT (LOCF) reported separately	Reasons listed	Specifics not disclosed	1 year
Foster^b^	2003	caloric restriction in low fat group	obese	Weight	Random number generator	Actual data, not imputed	Specifics not disclosed	Specifics not disclosed	1 year
Foster^b^	2010	caloric restriction in low fat group	BMI = 30–40	Weight	Random number generator	ITT reported (sensitivity analysis with completer data not significantly different)	Reasons listed	Weight at **Δ** = 3%	2 years
Gardner^a^	2007	no caloric restriction in Atkins or Ornish	women 25–50 yo, BMI = 27–40	Weight	Blocked, disclosed via envelopes	ITT (LOCF) reported (sensitivity analysis with completer data not significantly different)	Reasons listed	Weight at **Δ** = 2.7kg	1 year
Lean	1997	1200 kcal/day in each group	BMI≥25	Weight	Specifics not disclosed	ITT (LOCF) reported; also set deltas = 0 where no f/u data available	Specifics not disclosed	Specifics not disclosed	6 months
Lim^a^	2010	1548 kcal/day in each group	BMI = 28–40	Weight & CV risk factors	Stratified	Modeled to use partial data, rather than ITT or LOCF	Reasons listed	Weight at **Δ** = 1kg	15 months
Meckling	2004	restriction in both LoFAT & LoCHO	BMI>25 with dietary intake of >4000 kJ/d	Weight, body comp & lipids	Specifics not disclosed	Appears to be completer results from text	Specifics not disclosed	Specifics not disclosed	10 weeks
Ruth	2013	500 kcal deficit target in each group	BMI 29–45	Weight loss, with focus on adipose tissue inflammation	Randomization blocked	Completers reported	Reasons listed	Specifics not disclosed	12 weeks
Truby^a,b^	2006	None	BMI = 27–40	Weight and body fat	Stratified	For weight ITT (LOCF) with lipids reported for completers	Reasons listed	Weight at **Δ** = 4kg	6 months
Volek	2009	not instructed to reduce calories in either group (both did)	BMI>25	Cardiovascular risk factors	Specifics not disclosed	All completed	All completed	Specifics not disclosed	12 weeks
Yancy	2004	LoFAT with caloric restriction	BMI = 30–60 & abnormal lipids	Weight & lipids	Computer generated	Missing data imputed	Reasons listed	Specifics not disclosed	6 months

**Table 2:** Randomized clinical trials included in the meta-analyses: population characteristics.

Lead Author	Year	Minimum CHO Intake (g/d)	Minimum FAT Intake (%kcal/d)	% Men	n	% Complete LoCHO	% Complete LoFAT	ΔWeight LoCHO (95%CI)	ΔWeight LoFAT (95%CI)
Bradley	2009	94	20.0%	38%	27	86%	92%	-7.4 (-10.9, -3.9)	-6.5 (-10.1, -2.9)
Brehm	2003	41.1	28.0%	0%	53	85%	74%	-8.5 (-8.9, -8.1)	-3.9 (-4.3, -3.5)
Brehm	2005	48.3	29.0%	0%	50	80%	80%	-9.8 (-11.2, -8.4)	-6.1 (-7.9, -4.4)
Brinkworth	2009	19.8	27.0%	25%	118	54%	63%	-14.5 (-15.1, -13.9)	-11.5 (-11.9, -11.1)
Dansinger^a^	2005	68	17.1%	52%	80	53%	50%	-3.9 (-6.5, -1.3)	-6.6 (-10.7, -2.5)
de Luis	2012	120	25.1%	26%	305	100%	100%	-3.4 (-4.4, -2.4)	-4.1 (-5.1, -3.1)
Flechter-Mors	2010	114	29.4%	20%	110	56%	89%	-11.8 (-14.2, -9.4)	-6.9 (-8.8, -5.0)
Foster^b^	2003	20	25.0%	32%	63	61%	57%	-7.3 (-10.5, -4.1)	-4.5 (-8.3, -0.7)
Foster^b^	2010	20	30.0%	32%	307	58%	56%	-6.3 (-8.0, -4.6)	-7.4 (-9.1, -5.7)
Gardner^a^	2007	61.1	21.1%	0%	153	88%	78%	-4.7 (-6.1, -3.3)	-2.2 (-3.7, -0.7)
Lean	1997	110	20.5%	0%	110	81%	84%	-6.8 (-8.4, -5.2)	-5.6 (-7.1, -4.1)
Lim^a^	2010	56.9	12.5%	20%	60	57%	60%	-2.9 (-5.2, -0.6)	-2.1 (-4.3, 0.1)
Meckling	2004	59	17.9%	29%	31	100%	100%	-7.0 (-10.0, -4.0)	-6.8 (-10.0, -3.6)
Ruth	2013	39.4	25.1%	11%	55	62%	58%	-7.1 (-9.3, -5.0)	-5.3 (-7.6, -2.9)
Truby^a^ ^,^ ^b^	2006	49.6	26.0%	27%	115	70%	71%	-8.9 (-10.5, -7.3)	-8.8 (-10.4, -7.2)
Volek	2009	44.8	24.4%	50%	40	100%	100%	-10.2 (-12.9, -7.5)	-5.2 (-8.0, -2.4)
Yancy	2004	29.5	29.3%	23%	120	75%	57%	-12.0 (-14.4, -9.6)	-6.5 (-8.7, -4.3)

^a^ 3–5 arms in study, low carbohydrate compared to low fat

^b^ multicenter trial

#### Demographics

The characteristics of randomized controlled trials are presented in Table 1 with baseline characteristics of the population in Table 2. Study duration ranged from 8 weeks to 24 months with mean duration of 35.1 weeks (95% CI: 21.7, 48.4; median = 24 weeks). Three studies enrolled women only. The treatment groups were well balanced with similar number of subjects in the 2 randomized groups completed the study – 74.6% in the LoFAT diet and 74.4% in the LOCHO diet group (Table 3). There were, however, imbalances in drop out rates among the diet groups across individual trials (Table 2).

**Table 3:** Baseline characteristics of population, characterization of dietary interventions, adherence and effect on weight.

	Low CHO	Low FAT	
	Mean (95% CI)	Mean (95% CI)	p
***Baseline Characteristics***			
# Patients	895	902	
Age, years	43.5 (41.2, 45.8)	43.9 (41.9, 45.9)	0.77
% Men	23.3 (14.8, 31.8)	22.1 (13.6, 30.6)	0.83
Weight, kg	94.4 (91.6, 97.3)	94.1 (91.2, 96.9)	0.85
BMI kg/m^2^	34.0 (33.1, 34.8)	33.6 (32.7, 34.5)	0.55
Total Cholesterol, mg/dL	210 (200, 221)	207 (197, 218)	0.68
HDL-C, mg/dL	50.0 (47.3, 52.7)	49.9 (47.5, 52.3)	0.96
LDL-C, mg/dL	132 (121, 143)	130 (120, 140)	0.73
TG, mg/dL	144 (131, 156)	142 (127, 158)	0.86
Systolic BP, mmHg	127 (123, 130)	127 (123, 130)	0.92
***Baseline Risk Scores***			
Risk: White, lower-risk %	1.19 (0.90, 1.48)	1.22 (0.84, 1.60)	0.88
Risk: White, higher-risk %	4.88 (4.01, 5.74)	4.90 (3.89, 5.92)	0.97
Risk: African-American, lower-risk %	1.81 (1.35, 2.26)	1.85 (1.24, 2.46)	0.90
Risk: African-American, higher-risk %	6.38 (4.98, 7.78)	6.48 (4.71, 8.25)	0.93
***Dietary Interventions***			
% Complete	74.4 (65.6, 83.2)	74.6 (65.7, 83.5)	0.97
Energy, kcal/d	1504 (1386, 1622)	1449 (1367, 1531)	0.42
Carbohydrate g/d	60 (44, 76)	205 (186, 225)	< 0.00001
Protein, g/d	106 (96, 116)	70 (64, 76)	< 0.00001
Fat, g/d	90 (77, 104)	37 (32, 42)	< 0.00001

#### Interventions

In trials with randomization to one of several different diets; only data for the LoCHO and LoFAT groups were extracted.[18,23,25,28] Two trials used the Ornish diet for the LoFAT intervention.[18,23] The magnitude of carbohydrate and fat restrictions were calculated from the time of lowest daily intake (Table 2), at which time the average carbohydrate intake was 145 g/day lower in the LoCHO vs. LoFAT group. The LoFAT group averaged lower protein intake (by 36 g/day) and lower fat intake (by 53 g/day) with an average minimum fat intake of 24% of daily energy (95% CI: 21, 27). At the time of the strictest carbohydrate restriction for the LoCHO group, the total caloric intake was not significantly different between groups (1504 vs. 1449 kcal/day for LoCHO and LoFAT, respectively).

### Effect on Weight Loss

#### Frequentist Meta-Analysis

**Fig 2:**
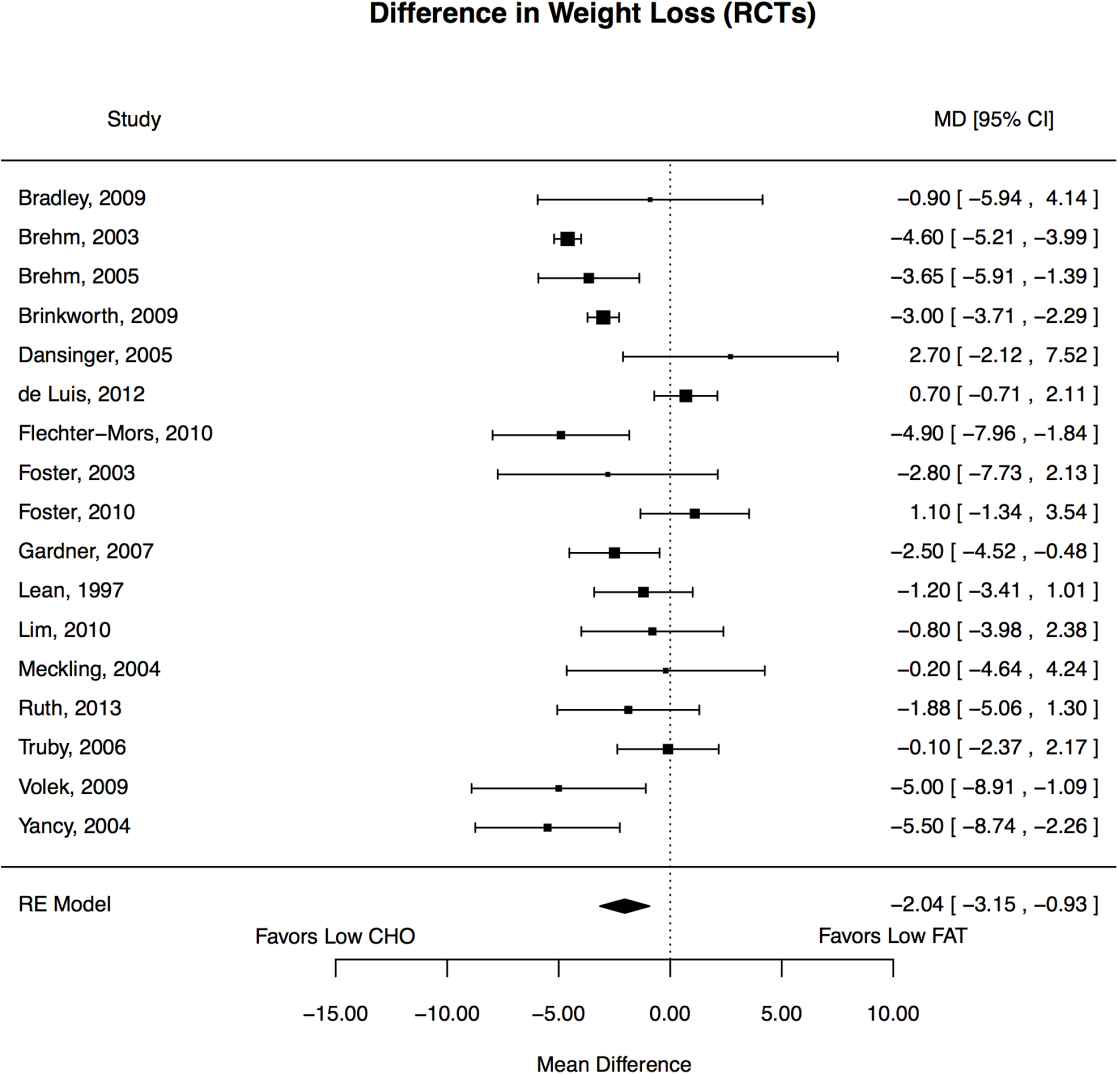
Forest plot of effects of diet on weight in the overweight and obese.

Of the 17 trials, 7 reported statistically significant reductions in weight favoring LoCHO diet and none favored LoFAT diet. (Fig 2) The weighted mean changes (baseline minus end-of-treatment) in outcomes were −7.8 versus −5.9 kg for body weight. Compared with participants on LoFAT diets, those on LoCHO diets experienced statistically significantly greater reduction in body weight (pooled mean net change, -2.0 kg, 95% CI: -3.1, -0.9). Weight decreased from 94.8 (95% CI: 91.6, 97.3) to 86.6 kg (95% CI: 83.6, 89.6) with LoCHO (p<0.0001). With LoFAT, weight decreased from 94.1 (95% CI: 91.2, 96.9) to 88.2 kg (95% CI: 85.4, 90.9 (p<0.0001). LoCHO significantly more favorably affected secondary outcomes of changes in HDL-C and triglycerides while LoFAT significantly more favorably affected total cholesterol and LDL-C. Effects on systolic blood pressure trended to favor LoCHO (p = 0.08). (Table 4)

**Table 4:** Frequentist and Bayesian meta-analyses of within group and between group differences of dietary interventions on metabolic parameters.

	Frequentist Analysis						Bayesian Analysis		
	Within Group Mean Differences				Between Group Differences ^a^		Between Group Differences ^a^		
	Low CHO		Low FAT						
	Mean (95% CI)	p	Mean (95% CI)	p	Mean (95% CI)	p	Mean (95% CrI	Probability LoCHO Superior	Probability LoFAT Superior
BMI kg/m^2^	-2.8 (-3.3, -2.2)	< 0.0001	-2.1 (-2.5, -1.7)	< 0.0001	-0.7 (-1.1, -0.3)	0.0016	-0.6 (-1.5, 0.3)	90.1%	
Cholesterol (mg/dl)	-4.2 (-9.4, 1.1)	0.11	-13.8 (-21.6, -5.9)	0.002	9.1 (2.6, 15.7)	0.006	9.6 (2.7, 16.4)		99.7%
HDL-C (mg/dl)	4.4 (2.3, 6.5)	0.0004	-1.0 (-3.2, 1.2)	0.35	5.1 (3.5, 6.7)	< 0.0001	5.4 (3.5, 7.2)	> 99.9%	
LDL-C (mg/dl)	-1.8 (-6.1, 2.6)	0.39	-10.9 (-17.3, -4.4)	0.0025	8.6 (3.6, 13.7)	0.0008	9.1 (3.0, 15.2)		99.8%
TG (mg/dl)	-41.1 (-54.7. -27.5)	< 0.0001	-11.3 (-18.8, -3.7)	0.006	-28.8 (-39.1, -18.5)	< 0.0001	-29.8 (-37.0, -22.6)	> 99.9%	
Systolic BP (mmHg)	-6.7 (-9.0, -4.3)	< 0.0001	-4.4 (-7.2, -1.5)	0.006	-1.7 (-3.5, 0.2)	0.08	-2.3 (-4.4, -0.2)	98.2%	

^a^ Between group differences as (LoCHO—LoFAT), positive mean value for between group differences reflects greater drop in LoFAT & negative value reflects greater drop in LoCHO.

#### Bayesian Meta-Analysis

The results of the Bayesian hierarchical modeling were generally similar to those based on the frequentist approach. The Bayesian credible intervals are relatively wider than the frequentist confidence intervals due to additional variability accounted for by the former. LoCHO diet yielded greater reduction in body weight loss than with LoFAT diet (mean difference: -1.8 kg, 95% CI: -3.1, -0.5).

#### Bayesian Probability

**Fig 3:**
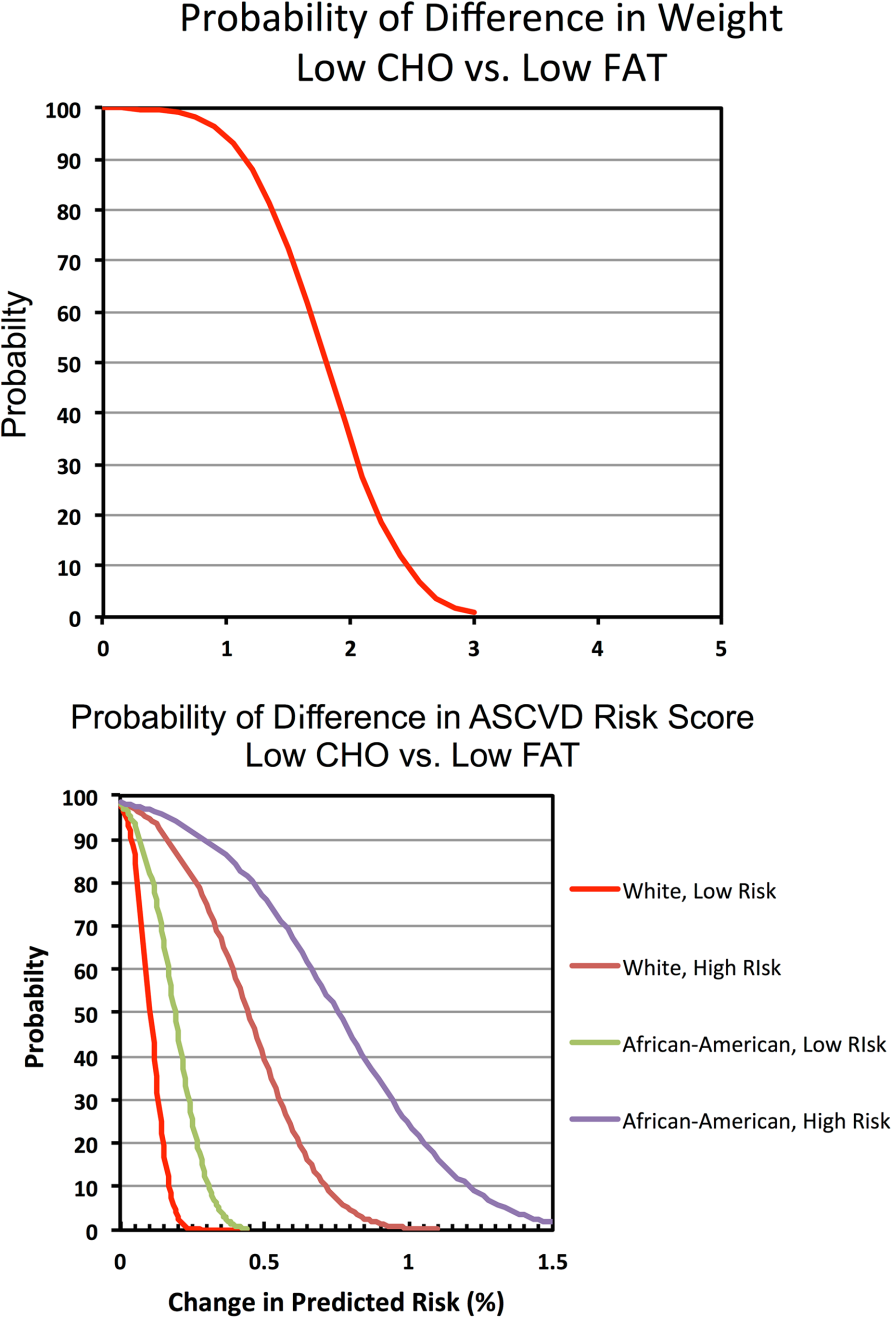
Bayesian probabilities for mean differences in (a) weight loss and (b) estimated 10-year ASCVD risk scores.

An advantage of the Bayesian approach is the computation of posterior probability for any given treatment effect and graphic representation of these probabilities across a range of treatment effects as shown. (Fig 3) The results indicate a 100% chance that the weight loss is >0 kg with both LoCHO and LoFAT diets. Moreover, the probability for benefit falls as the threshold for benefit increases. (Fig 3A) The results show that there is >99.9%, 89% and 38% chance that the mean weight loss on LoCHO diet exceeds the weight loss on LoFAT diet by greater than 0, 1, and 2 kg, respectively. Thus, although a conventional frequentist analysis shows that LoCHO diet is associated with a statistically significant reduction in outcome (>0), Bayesian analysis helps clarify the likelihood of any given magnitude of difference, thereby providing insights regarding the clinical importance of benefit or harm. LoCHO was >99.9% likely to be associated with superior effects on HDL-C and triglycerides than LoFAT, while LoFAT was >99.7% likely to be associated with superior effects on total cholesterol and LDL-C. (Table 5)

**Table 5:** Frequentist and Bayesian meta-analyses of within group and between group differences of dietary interventions on predicted ASCVD risk for each subgroup.

Predicted Risk, % (Frequentist)		White, lower-risk	White, higher-risk	African-American, lower-risk	African-American, higher-risk
Low CHO (within group)	Baseline	1.19 (0.90, 1.48)	4.88 (4.01, 5.74)	1.81 (1.35, 2.26)	6.38 (4.98, 7.78)
	Outcome	0.99 (0.72, 1.26)	4.03 (3.17, 4.88)	1.50 (1.04, 1.97)	5.08 (3.63, 6.53)
	Mean (95% CI)	-0.20 (-0.27, -0.13)	-0.85 (-1.10, -0.60)	-0.30 (-0.41, -0.19)	-1.30 (-1.72, -0.88)
	p	< 0.0001	< 0.0001	< 0.0001	< 0.0001
Low FAT (within group)	Baseline	1.22 (0.84, 1.60)	4.90 (3.89, 5.92)	1.85 (1.24, 2.46)	6.48 (4.71, 8.25)
	Outcome	1.13 (0.75, 1.51)	4.49 (3.44, 5.54)	1.74 (1.11, 2.37)	5.94 (4.13, 7.74)
	Mean (95% CI)	-0.10 (-0.17, -0.02)	-0.41 (-0.72, -0.10)	-0.11 (-0.24, 0.02)	-0.54 (-1.04, 0.04)
	p	0.01	0.01	0.086	0.036
Between Group Differences	Mean (95% CI)	-0.10 (-0.20, -0.01)	-0.44 (-0.83, -0.06)	-0.19 (-0.35, -0.03)	-0.76 (-1.38, -0.14)
	p	0.03	0.025	0.02	0.02
**Predicted Risk, % (Bayesian)**					
Low CHO (within group)	Baseline	1.18 (0.89, 1.48)	4.89 (3.98, 5.81)	1.80 (1.33, 2.29)	6.38 (4.88, 7.84)
	Outcome	0.98 (0.70, 1.27)	4.01 (3.11, 4.91)	1.49 (1.00, 1.98)	5.03 (3.50,6.55)
	Mean (95% CrI)	-0.20 (-0.61, 0.21)	-0.88 (-2.15, 0.40)	-0.31 (-1.01, 0.37)	-1.35 (-3.48, 0.75)
	Probability Reduced Risk	84.1%	91.6%	82.1%	89.9%
Low FAT (within group)	Baseline	1.15 (0.79, 1.52)	4.83 (3.80,5.88)	1.80 (1.18, 2.42)	6.37 (4.58, 8.24)
	Outcome	1.05 (0.68, 1.42)	4.39 (3.34, 5.49)	1.67 (1.05, 2.30)	5.78 (3.99,7.66)
	Mean (95% CrI	-0.10 (-0.61, 0.40)	-0.44 (-1.94, 1.00)	-0.13 (-1.00, 0.73)	-0.59 (-3.19, 1.94)
	Probability Reduced Risk	66.7%	72.5%	62.1%	68.2%
Between Group Differences	Mean (95% CrI)	-0.11 (-0.21, 0.01)	-0.44 (-0.84, 0.02)	-0.19 (-0.37, -0.12)	-0.76 (-1.44, -0.06)
	Probability LoCHO Superior	98.1%	98.1%	98.1%	98.3%

### Effect on ASCVD Risk

#### Predicted Risk via Pooled Cohort Equations

Data from 15 trials were available to estimate 10-year ASCVD risk for each of the 4 subsets, White and African-American populations, each with lower- and higher-risk assumptions.

#### Frequentist Meta-Analysis

Compared with baseline, both LoCHO and LoFAT were associated with reductions in estimated 10-year risk score for ASCVD. (Table 5) In all 4 subsets benefits of LoCHO were highly statistically significant, with benefit apparent as well in the LoFAT diet (within which there were statistically significant benefits at p<0.05 for all subsets except lower-risk African-Americans [p = 0.086]). LoCHO significantly reduced predicted risk of ASCVD events compared to LoFAT in all 4 subsets (p ≤0.03), with LoCHO associated with an absolute difference in risk ranging from 0.1% in lower-risk Whites to 0.76% in higher-risk African-Americans.

#### Bayesian Meta-Analysis

By Bayesian modeling, the likelihood that LoCHO was associated with greater improvement in predicted risk compared with LoFAT diet was at least 98.1%. The probability of reduction in ASCVD risk with LoCHO diet ranged from 82% in lower-risk African-Americans to 92% in higher-risk Whites, while LoFAT was associated with reduction in risk that ranged from 62% in lower-risk African-Americans to 72% in higher-risk Whites. (Table 6)

**Table 6:** Sensitivity analysis for effects of diet interventions on weight using frequentist meta-analysis method.

	Trials	Frequentist		Bayesian		
Group	(n)	Change in Weight (95% CI)	P	Change in Weight (95% CI)	Pr>0 of Any Weight Loss
Duration					
<6 mo	6	-1.7 (-3.7, 0.2)	0.086	-1.9 (-4.5, 0.8)	94.0%
= 6 mo	4	-2.8 (-5.3, -0.4)	0.0253	-2.7 (-8.7, 3.0)	98.0%
≥1 yr	7	-1.7 (-3.5, 0.01)	0.051	-1.4 (-4.3, 1.5)	85.2%
% Drop Out					
Lowest Tercile	6	-1.1 (-2.9, 0.7)	0.04	-0.6 (-3.7, 2.3)	69.1%
Mid Tercile	6	-3.3 (-5.0, -1.5)	0.0002	-3.3 (-6.3, -0.3)	98.2%
Highest Tercile	5	-1.4 (-3.5, 0.7)	0.19	-1.7 (-5.2, 1.9)	87.8%
% Male					
Lowest Tercile	7	-3.0 (-4.3, -1.8)	<0.0001	-2.6 (-4.4, -0.8)	99.4%
Mid Tercile	5	-1.6 (-3.8, 0.6)	0.16	-1.6 (-5.8, 2.7)	82.6%
Highest Tercile	5	-0.9 (-3.6, 1.9)	0.52	-1.0 (-5.7, 3.7)	71.7%
Population Size					
Lowest Tercile	6	-3.5 (-4.9, -2.1)	< 0.0001	-2.5 (-5.2, 0.1)	97.1%
Mid Tercile	6	-1.3 (-3.1, 0.5)	0.15	-1.2 (-4.4, 2.1)	81.1%
Highest Tercile	5	-1.7 (-4.0, 0.5)	0.13	-1.8 (-6.4, 2.8)	84.2%
Reporting Method					
Completers	11	-2.4 (-3.9, -1.0)	0.001	-2.0 (-3.9, -0.1)	98.0%
Intention to Treat	6	-1.4 (-3.0, 0.3)	0.1	-1.5 (-4.4, 1.4)	88.9%
SD of Change in Weight for Meta-Analysis					
SD reported in publication	12	-2.4 (-3.6, -1.1)	0.0002	-2.3 (-3.8, -0.9)	99.7%
SD imputed	5	-0.8 (-3.1, 1.5)	0.5	-0.7 (-5.1, 3.6)	67.5%
Difference in Carbohydrate Intake Between Groups (LoCHO–LoFAT)					
Lowest Tercile	5	-2.7 (-4.8, -0.6)	0.01	-2.7 (-6.5, 1.1)	94.3%
Mid Tercile	5	-1.5 (-3.2, 0.3)	0.09	-0.5 (-4.3, 3.0)	62.4%
Highest Tercile	4	-3.7 (-5.3, -2.0)	< 0.0001	-3.5 (-7.8, 0.7)	96.2%
Difference in Calorie Intake Between Groups (LoCHO–LoFAT)					
Lowest Tercile	5	-3.0 (-5.2, -0.8)	0.007	-3.1 (-7.0, 0.8)	95.5%
Mid Tercile	5	-2.9 (-4.0, -1.7)	< 0.0001	-2.9 (-6.7, 0.9)	95.0%
Highest Tercile	5	-0.4 (-1.9, 1.0)	0.56	-0.2 (-3.1, 2.6)	56.4%

#### Bayesian Probability

The likelihood of a reduction in the predicted risk score by at least 0.1% with LoCHO relative to LoFAT ranged from 55–84% in lower-risk to 95–96% in higher-risk subsets, while the likelihood of a reduction of at least 0.15% ranged from 19–67% in lower-risk to 92–95% in higher-risk subsets. (Fig 3B)

#### Sensitivity analysis

**Fig 4:**
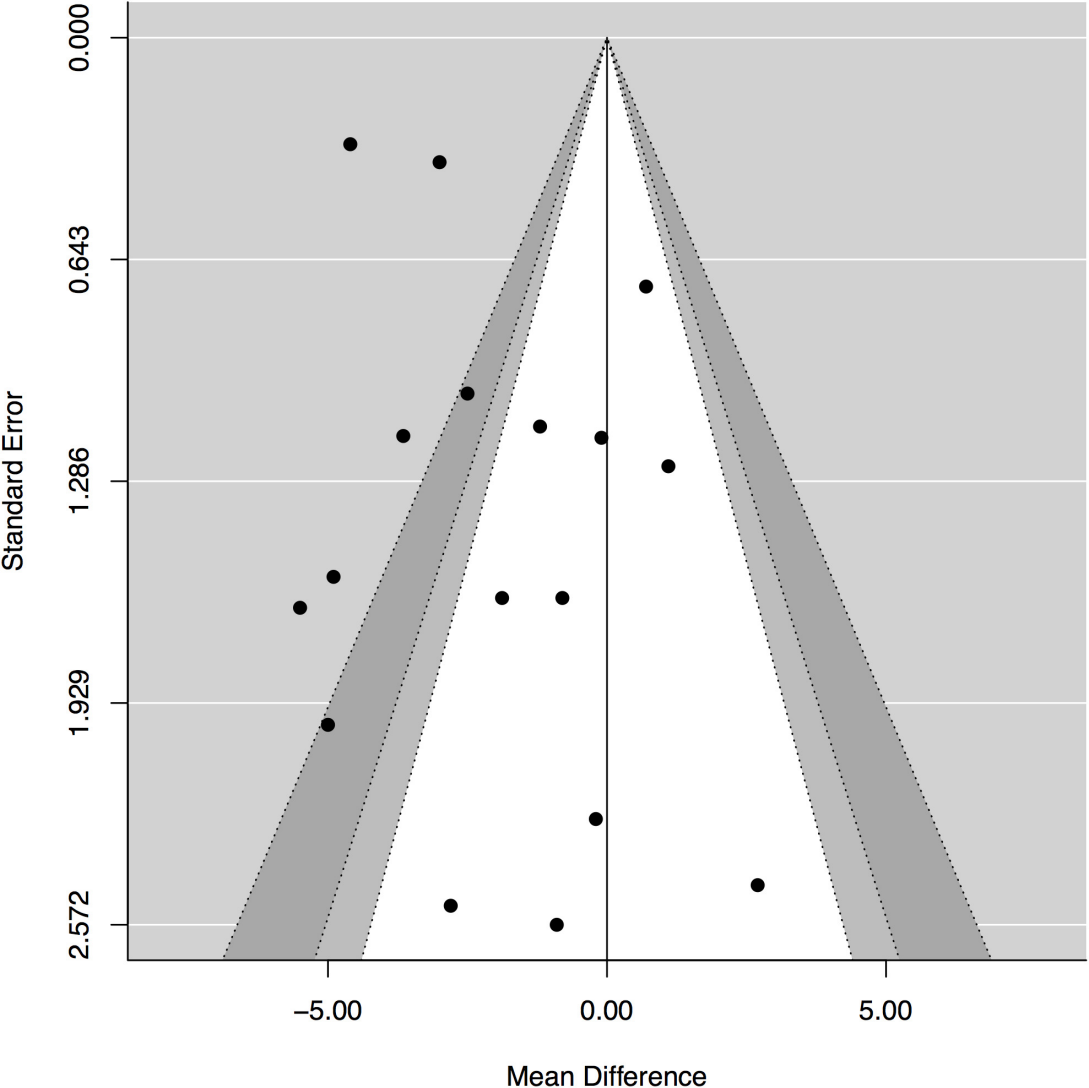
Funnel plot of the effect on weight as relates to the size/precision of the results from each trial.

Funnel plot suggested the possibility of bias for the results on changes in weight. (Fig 4) Subgroup analyses by gender, diabetic status, drop out rate and extent of missing data did not identify meaningful explanations. (Table 6) We repeated the meta-analysis using fixed effects model. The results indicate a numerically greater advantage of LoCHO on weight loss compared with weight loss observed using the random effects model (mean difference: -3.1, 95% CI: -3.5, -2.7, p<0.0001). This indicates that weight loss in smaller studies (given a relatively greater weight in random effects model) is unlikely to explain funnel plot asymmetry.[31]

## Discussion

Our trial-level meta-analysis of randomized controlled trials comparing LoCHO diets with LoFAT diets in strictly adherent populations demonstrates that each diet was associated with significant weight loss and reduction in predicted risk of ASCVD events. However, LoCHO diet was associated with a numerically modest but statistically significantly greater improvement in weight loss and reduction in predicted ASCVD risk. These results provide strong justification for a reevaluation of LoCHO diets that are currently not endorsed by the dietary guidelines for the overweight and obese.

These analyses provide insights for clinicians and public health policy makers. The use of Bayesian hierarchical modeling provides an estimate of the likelihood of achieving a desired degree of weight loss, which can be integrated into decision-making process. Further, the meta-analysis evaluates the cardiovascular risk of these dietary approaches beyond the crude estimates possible from focus on an individual parameter such as HDL-C or LDL-C. The NHLBI Pooled Cohort Equation-derived score is a global index of ASCVD risk recommended for use in clinical settings by the 2013 AHA/ACC prevention guidelines.[10,32] This global risk score has been shown to be a more powerful predictor of risk than LDL-C alone,[10] yet several studies suggest these equations might overestimate risk.[33–37] Nonetheless, our results permit an inference of reduced risk with both dietary interventions relative to baseline and greater improvement in predicted risk scores with LoCHO compared with LoFAT. Over the past several decades, LoFAT diets have been recommended to the public for weight loss primarily because of their beneficial associations with metabolic risk factors and cardiovascular events.[1] Our study suggests that LoCHO diets might provide an alternative approach for weight reduction with effects on metabolic risk factors that suggest a significant reduction in ASCVD risk associated with the intervention of LoCHO diets.

Our results are consistent with the effects reported in prior meta-analyses of LoCHO diets on weight, lipid profiles and blood pressure.[38,39] In the analysis by Bueno et al, which included trials with stricter carbohydrate restriction than the trials selected in our analysis (including requirement for presence of ketosis),[38] LoCHO diet was associated with greater weight loss (-0.91 kg, 95% CI: -1.65, -0.17) than LoFAT comparators. Hu et al. reported a numerical, but not statistical, difference in weight loss favoring LoCHO diet (-1.0 kg, 95% CI: -2.2, 0.2).[39] Two potential reasons could account for the differences in the estimated magnitude of the weight loss achieved in that study compared with our report. First, the definition of LoCHO diet was relatively less stringent in Hu et al. with daily intake not exceeding 45% of total energy, (approximately the equivalent of over 200 g/d of carbohydrates in 1800 kcal/d intake compared with 120 g/d in the trials included in our report). Typical LoCHO diets for weight loss restrict carbohydrate to less than 20–30% of daily energy intake.[40] Second, follow up of trials in our study was shorter with 6 trials reporting follow-up <6 months compared with requirement for ≥6 months follow up for trials included in the Hu et al. report[39] or ≥12 months for the trials included in the report by Bueno et al.[38] A previous meta-analysis by Santos et al. suggested an attenuation of treatment effect at longer follow up (LoCHO diets were associated with numerically smaller weight loss advantage at 24 months relative to shorter observation periods).[41] Our sensitivity analysis also confirm these results—mean weight loss in trials of ≥12 months follow up was about 39–48% lower than those with follow up of 6 months, although weight loss advantage of LoCHO diet was similar in the shortest and the longest follow up trial in our analysis. (S2 File) In a recent report by Johnston et al, several dietary interventions were shown to be effective for weight management with small, but not clinically relevant, differences in weight loss between individual diets.[42] The report was based on a larger number of studies (n = 48 trials, 7286 individuals) with broader inclusion criteria including populations with co-morbidities, no requirement for strict adherence to the intervention, and limited follow-up (up to 12 months). The largest weight loss was associated with LoCHO diet: 8.73 kg and 7.25 kg at 6-month and 12-month follow up, respectively. Weight loss with LoFAT diet was 7.99 kg and 7.27 kg at these time points. These findings are consistent with our results and highlight the importance of policy-makers in reevaluating the role of LoCHO diets for weight management, particularly relative to the current endorsement for LoFAT diets by professional society guidelines.

Our study has several limitations. First, patient-level data for each study was not available. Compared to trial summary data, patient-level data permits evaluation of each study’s quality and eligibility for inclusion in a meta-analysis, allows for confirmation of study outcomes and facilitates evaluation of the consistency of treatment effects across important subgroups.[43] Second, losses to follow-up were substantial, including imbalances in dropout rates amongst the randomized intervention groups leading to potential for informative censoring. Half of the studies included in our meta-analysis had completion rates less than 73%, while 25% had less than 59% completion rate (minimum 52%) and 25% had at least 83% completion rate. However, the sensitivity analysis suggested a nonsignificant influence of studies with a low completion rate on the overall study results. Third, publication bias may be responsible for the significant differences in primary and secondary endpoints, based on the observation of asymmetry of the funnel plot and significant heterogeneity across trials.[31] Analysis of the outcome by subgroups (such as trial duration, size and population studied) failed to demonstrate an explanation for the asymmetry, though the small number of trials and lack of patient-level data hinders the ability to understand clearly the basis for this observation. However, because fixed effects model for the meta-analysis reveals a numerically higher estimate of benefit than the random effects model, the asymmetry is unlikely to be based on smaller trials carrying excess weight in the analysis. Fourth, the estimated ASCVD risk scores were based on post hoc analysis utilizing assumptions (ethnicity, tobacco use and blood pressure medications) that are not verifiable. By calculating the risk based on the most extreme assumptions possible for the lower-risk and higher-risk subgroups, the true effect is constrained within these two extremes. Fifth, underlying mechanisms that may account for differences in weight loss by diet are not discernible from this report, thus the impact on risk factors cannot be untangled from diet-induced weight loss versus specific metabolic effects. Sixth, the tools used to assess dietary intake rely on subject recall, a source of bias. However, we do not expect a differential effect of the recall bias on the dietary intervention groups. Seventh, results were not adjusted for multiple comparisons. However, given the extremely robust P values, this adjustment is unlikely to materially alter the principal results. Eighth, the data available from the component studies of this meta-analysis did not consistently disclose the quality of fat in the diets, i.e., the proportions that were saturated, unsaturated or polyunsaturated. Such an analysis would be useful in light of the recent AHA/ACC Guideline recommending a shift from saturated fats for reduction of cardiovascular risk,[44] to learn whether such a recommendation is warranted as the obese and overweight contemplate weight management strategies. Finally, none of the trials were designed to examine long-term effects on cardiovascular outcomes, and thus, the inferences on risk are based on risk prediction and not actual events.

There are also several strengths in the present study. We conducted this meta-analysis following a stringent protocol. The data were abstracted using a standard abstraction form and entered into a database via double entry. The studies that we used were all randomized controlled trials, which are subject to fewer biases than observational studies and are the gold standard for evaluating the effects of an intervention. This meta-analysis had a sample size of 1,797, which provided the power to detect statistically significant mean differences, assess publication bias, and conduct sensitivity and subgroup analyses. Moreover, we included trials over a wide range of treatment durations to evaluate both short-term and long-term changes in weight loss and metabolic risk factors. Finally, we included Bayesian modeling that allows incorporation of additional sources of uncertainty and quantification of probability of treatment effect of any magnitude, including clinically relevant differences. The concordance of the meta-analytic results based on the frequentist and Bayesian approaches lends additional confidence to our conclusions.

These findings have important clinical and public health implications. Over the past several decades, guideline recommendations have emphasized LoFAT diets over other dietary interventions for weight loss and modification of cardiovascular risk factors. Our study suggests that LoCHO diets might provide a viable, and arguably a preferred, option for achieving this goal. While the mean difference in weight loss between diets appears somewhat modest, the possibility of meaningful public health impact should not be dismissed, particularly if such an effect could be sustained long-term in a large population. Ideally, further studies are warranted to clarify the role of these diets on intermediate risk markers and to assess whether these changes would translate into long-term reduction in cardiovascular risk. However, given modest differences in intermediate risk markers among dietary interventions, attenuation of treatment effects over time,[25,40,41] and the challenges of maintaining long-term adherence with dietary interventions, an adequately powered mega trial enrolling tens of thousands of patients followed for a long period would be required. It is unlikely such a trial would be feasible. Thus, policy decisions and clinical practice is likely to be driven by results of meta-analyses and systematic reviews, with availability of patient-level data yielding more robust inferences.[43]

In conclusion, this trial-level meta-analysis of 17 randomized controlled trials shows that both LoCHO and LoFAT diets are effective in reducing weight. However, LoCHO diet appears to achieve greater weight loss and reduction in predicted risk of ASCVD events compared with LoFAT diet. On the basis of these results, we suggest that dietary recommendations for weight loss should be revisited to consider this additional evidence of the benefits of LoCHO diets.

## Supporting Information

S1 File Pubmed Search Strategy. Search as performed on S/9/2014.
(DOCX)

S2 File Trial level data. Data are provided for individual trials, with “rx” representing LoCHO and “control” LoFAT diets, respectively.
(CSV)

S3 File R Code for data analysis. (TXT)

S4 File PRISMA 2009 Checklist. (DOCX)
