# Supplementary material for: Dietary Intervention for Overweight and Obese Adults: Comparison of Low-Carbohydrate and Low-Fat Diets. A Meta-Analysis
Source: PLoS One. 2015 Oct 20;10(10):e0139817. doi: 10.1371/journal.pone.0139817 (PMC4618935; doi:10.1371/journal.pone.0139817)
Supplement: S1 File — Search as performed on S/9/2014. (DOCX) [file pone.0139817.s001.docx]

Pubmed Search Strategy

Performed 09/09/2014

Search ((((((((overweight [mh]) OR obesity [mh]) OR adipose tissue [mh])) OR ((weight gain [mh]) OR weight loss [mh])) OR ((((body mass index [mh]) OR skinfold thickness [mh]) OR waist-hip ratio [mh]) OR body fat distribution [mh]))) AND (((((Diet, Fat-restricted [mh]) OR Diet, Carbohydrate-Restricted [mh])) OR ((Atkins diet) OR ((high-protein AND diet)))) OR ((((((((diet AND fat-restrict*))) OR ((diet* AND carbohydrate- restrict*))) OR ((diet AND fat-reduc*))) OR ((diet AND carbohydrate- reduc*))) OR ((diet AND low fat*))) OR ((diet AND low carbohydrat*))))) AND (((((((((randomized controlled trial [tiab]) OR controlled clinical tril [tiab]) OR randomi?ed [tiab]) OR placebo [tiab]) OR randomly [tiab])) OR ((meta-analysis [mh]) OR ((meta analy* OR metaanaly* OR meta? analy*)))) OR technology assessment, biomedical [mh]) OR ((hta OR health technology assessment OR (health technology AND assessment$))))

Result => 437 citations
